# Mesenteric venous thrombosis as a complication of appendicitis in an adolescent: A case report and literature review

**DOI:** 10.1097/MD.0000000000018002

**Published:** 2019-11-27

**Authors:** Seo Hee Yoon, Mi-Jung Lee, Se Yong Jung, In Geol Ho, Moon Kyu Kim

**Affiliations:** a Division of Pediatric Emergency Medicine, Department of Pediatrics, Severance Children's Hospital; b Department of Radiology, Severance Hospital, Research Institute of Radiological Science; c Division of Pediatric Cardiology, Congenital Heart Disease Center, Severance Cardiovascular Hospital, Department of Pediatrics; d Department of Pediatric Surgery, Severance Children's Hospital, Yonsei University College of Medicine, Seoul, Republic of Korea.

**Keywords:** adolescent, appendicitis, complication, mesenteric venous thrombosis, superior mesenteric vein thrombosis

## Abstract

**Rationale:**

Mesenteric venous thrombosis is an uncommon but potentially fatal condition that can cause bowel ischemia. It results from a systemic hypercoagulable state or abdominal infection draining into the portal venous system. Several cases regarding portomesenteric venous thrombosis as a complication of appendicitis were reported in adults, but there are far fewer reports in pediatric patients. The mortality rate of the condition is high if untreated, especially in children, reaching up to 50%.

**Patient concerns:**

A healthy 15-year-old male with no significant past medical history presented with right lower quadrant pain, lethargy, and fever. The computed tomography scan showed a focal thrombosis at the superior mesenteric vein branch and an inflamed appendix.

**Diagnoses:**

Mesenteric venous thrombosis complicating acute appendicitis.

**Interventions:**

Intravenous antibiotics along with anticoagulants and laparoscopic appendectomy

**Outcomes:**

After 1 month, a follow-up ultrasonography revealed full resolution of the thrombosis.

**Lessons:**

Appendicitis is one of the most frequently encountered causes of pediatric surgical emergencies; therefore, physicians should be conscious of mesenteric venous thrombosis as a possible complication of acute appendicitis, irrespective of whether patients have thrombophilic conditions or not.

## 1 Introduction

Mesenteric venous thrombosis is a rare cause of bowel ischemia, resulting from abdominal infection draining into the portal venous system (in diverticulitis, appendicitis, and cholecystitis), or in systemic prothrombotic states (smoking, obesity, hypertension, immobilization, surgery, trauma, oral contraceptives, pregnancy, deficiencies of coagulation factors, cancer, etc.).^[1–4]^ The diagnosis is frequently delayed because the presenting symptoms and physical examination and laboratory features are usually subtle and non-specific.^[5,6]^ The overall incidence of mesenteric venous thrombosis is estimated at 1 in 1000 emergency department admissions and accounts for 6% to 9% of all acute mesenteric ischemic events.^[1,7]^

There have been several reports regarding portomesenteric venous thrombosis as a complication of appendicitis in adults, but there are far fewer reports in children and adolescents. The mortality rate of the condition is high if untreated, especially in children, reaching up to 50%.^[8,9]^ Therefore, it is crucial for physicians to notice a mesenteric venous thrombosis as a possible complication of acute appendicitis, which is one of the most common surgical emergencies in pediatric patients.

Here we present a case of mesenteric venous thrombosis complicating acute appendicitis in a previously healthy adolescent and we also review the literature of the clinical features, management, and outcomes of the condition in pediatric patients. This study was approved by the Institutional Review Board of the Yonsei University Severance Hospital. Verbal informed consent was obtained from the patient for publication of the case details and the accompanying images. The verbal consent has been documented and will be made available on request.

## 2 Case presentation

A healthy 15-year-old male with no significant past medical history presented to the ED with a 2-day history of right lower quadrant (RLQ) pain, lethargy, and 1 day of fever. He had no known allergies. He was not taking any prescribed medications and denied cigarette smoking and use of alcohol. There was no family history of bleeding or clotting disorders.

At presentation, his vital signs included a temperature of 37.0°C, a pulse rate 98 beats/minute, a respiratory rate 16 breaths/minute, and a blood pressure of 109/57 mm Hg. On physical exam, there was significant RLQ tenderness without rebound tenderness, rigidity, or muscle guarding. Laboratory tests showed an elevated white blood cell count of 14,130/μl (neutrophils 95.4%), an aspartate aminotransferase level of 52 IU/L, a total bilirubin level of 2.2 mg/dl and a direct bilirubin level of 0.8 mg/dl, a high C-reactive protein level of 136.8 mg/L, a prolonged prothrombin time of 17.5 seconds (international normalized ratio [INR] 1.56), and an activated partial thromboplastin time (aPTT) of 33.5 seconds. Other laboratory test results were unremarkable.

**Figure 1:**
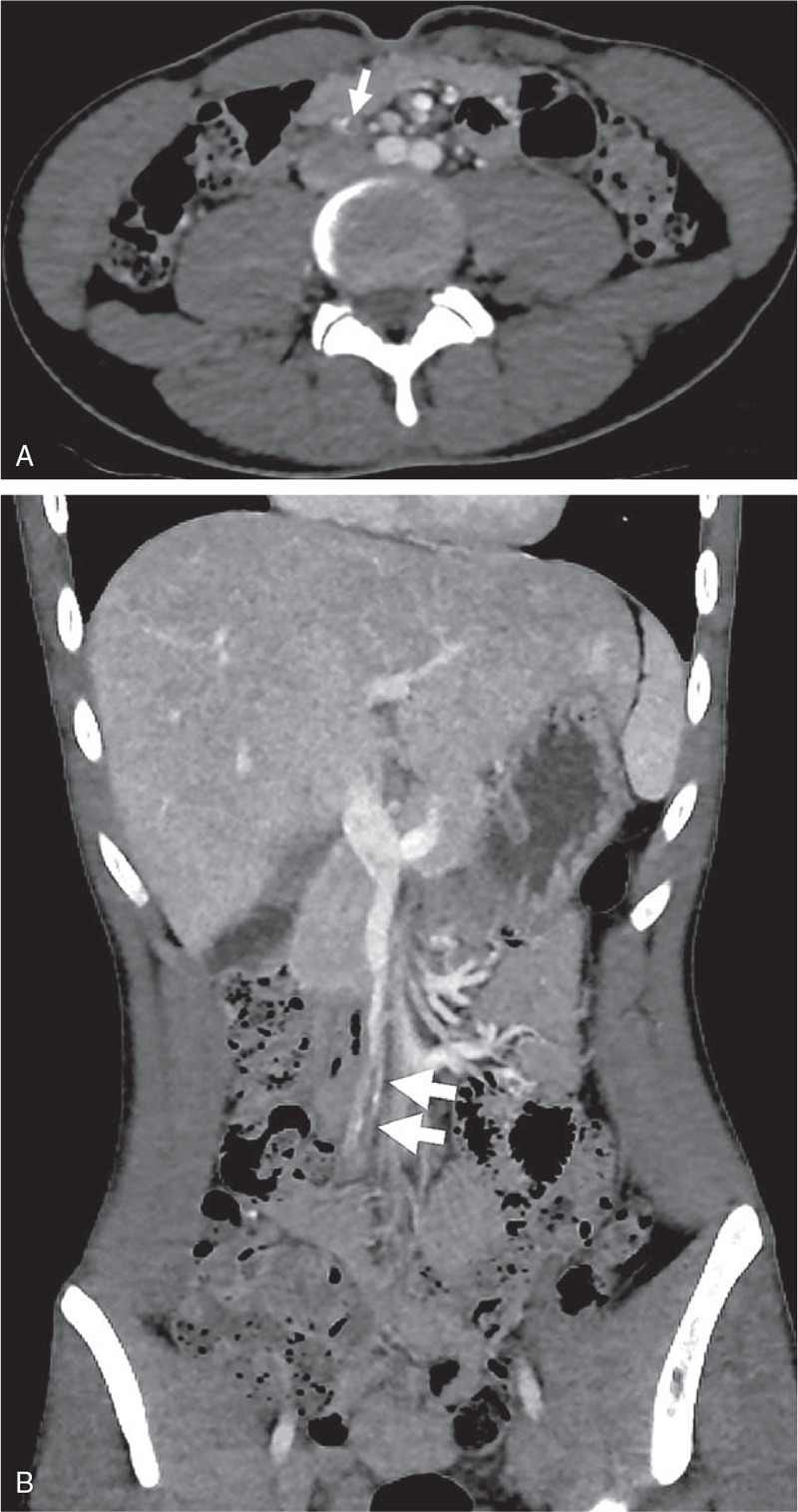
Computed tomography scan demonstrated a focal thrombotic occlusion of the superior mesenteric branch without associated bowel change (A)(B) (see arrow).

**Figure 2:**
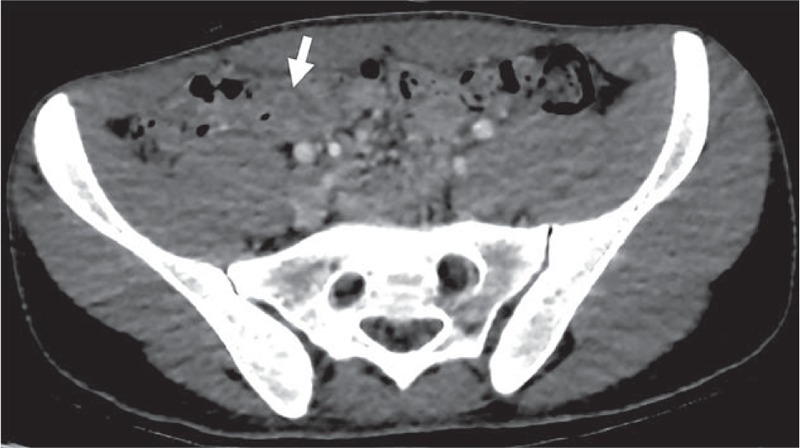
Focal fluid distension at the appendix tip and aeration at the proximal portion of the appendix (see arrow).

The computed tomography (CT) scan showed a focal thrombosis at the superior mesenteric vein (SMV) branch with patent distal and proximal flow without abnormal bowel changes (Fig. 1). Focal fluid distension at the appendix tip and aeration at the proximal portion of appendix was noted (Fig. 2). Otherwise the small and large bowel loops were not remarkable.

Additional coagulation tests were performed for measuring his coagulability status and then anticoagulation therapy with intravenous heparin was immediately started. The test results showed an elevated D-dimer level (963 mg/ml, normal 0–243) and low protein C activity (46%, normal 70–130). Other tests including protein S activity (64%, normal 62–154), fibrinogen functional level (382 mg/dl, normal 200–400), and antithrombin III level (84%, normal 80–120) were within their normal ranges. The patient received consultation from the Department of Hematology as prolonged INR with decreased protein C activity were thought to be a manifestation of non-overt disseminated intravascular coagulation from the intra-abdominal infection. After obtaining a blood culture, we also started intravenous antibiotics (ceftriaxone and metronidazole).

The patient's RLQ pain did not improve over the next 2 days; therefore, he was referred to the Pediatric Surgery Department and underwent laparoscopic appendectomy. The appendix was noted as being inflamed and swollen. There was no evidence of ischemic change of the intestine or active bleeding. Microorganisms were not found in the blood culture. INR and abnormal coagulation tests (except aPTT due to heparinization) were normalized before discharge. On hospital day 9, the patient was discharged without any surgical complications. Intravenous heparin was replaced with apixaban (oral factor Xa inhibitor, 2.5 mg twice daily for 3 months) at discharge. After 1 month, a follow-up ultrasound revealed full resolution of the SMV thrombosis.

## 3 Discussion

We present a healthy adolescent who was diagnosed with SMV thrombosis and appendicitis with no predisposing history. We found 19 reports of portomesenteric vein thrombosis complicating acute appendicitis in children and adolescents from 1979 to 2016 with ages ranging from 5 to 16 years (Table 1).^[2,10–26]^ The time from the initial symptom to diagnosis varied from a few days to 6 weeks. Delayed diagnosis occurred mostly due to the nonspecific presentation or symptoms which might to be attributed to the appendicitis itself. Concomitant thrombophilic conditions or a predisposing history were not found in most cases. Liver abscess was detected in 8 (42.1%) cases.^[10,13,14,20–22,25,26]^ Four cases (21.1%)^[2,15,17,23]^ underwent delayed appendectomy. The more recent half of the cases (9/19, 47.3%)^[2,16,19–24,26]^ were treated with anticoagulants from 3 months to 1 year. Two cases (10.5%) were found after appendectomy due to the vomiting^[16]^ and RLQ pain.^[18]^ The overall prognosis was good, but portal hypertension with bleeding esophageal varices occurred in 1 case,^[12]^ and 2 cases reported that the resolution of thrombosis was not complete at last follow-up visit.^[10,26]^

**Table 1:**
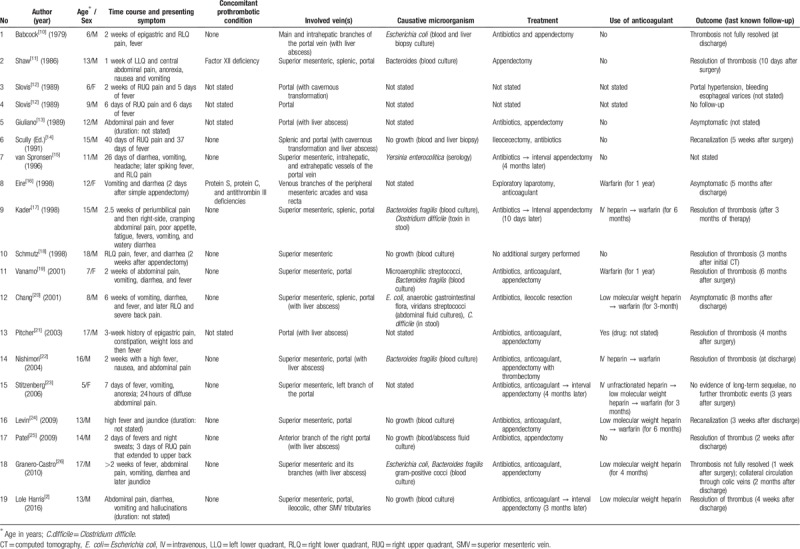
Patient demographics, symptoms, management of the 19 reported cases of portal/mesenteric venous thrombosis complicating appendicitis in children.

Laboratory tests might reveal leukocytosis, elevated liver enzyme levels, hyperbilirubinemia, and coagulopathy, but the results are not helpful for diagnosis, although they can be used for identifying the thrombophilic condition.^[1,27,28]^ In our case, the patient's laboratory examination revealed a mild elevation of aspartate aminotransferase and bilirubin with coagulopathy, but those abnormalities were normalized before discharge (aPTT was normalized after heparin discontinuation).

Blood and abdominal fluid cultures can be helpful in identifying the causative microorganisms in mesenteric vein thrombosis. In adult cases, *Escherichia coli, Klebsiella pneumoniae*, *Bacteroides fragilis, Proteus mirabilis* are commonly detected causative microorganisms.^[29,30]^ In pediatric cases, the causative microorganisms found in 42.1% (8/19) of cases included *Bacteroides fragilis*, *Escherichia coli, Yersinia enterocolitica,* and *Clostridium difficile*.^[10,11,15,17,19,20,22,26]^*Bacteroides fragilis* was the most common isolated organism (4/8, 50.0%).^[11,17,19,22,26]^ Physicians should consider these pathogens when initiating empirical antibiotic therapy.

A filling defect of the vessel was found in about 90% of cases in contrast-enhanced CT scans.^[1,31]^ The presence of locules of air within the SMV or portal venous system is another CT finding suggestive of thrombosis.^[32]^ CT is also helpful for identifying the primary infection source and other accompanying lesions such as a liver abscess.^[32,33]^ Doppler ultrasonography is the first-line study for the diagnosis of portal vein thrombosis and is mostly useful in the follow up of these cases as it was with our case.^[32,34]^

Mesenteric venous thrombosis can lead to bowel ischemia or infarction unless treated. Therefore, if mesenteric venous thrombosis is diagnosed, adequate treatment should be promptly initiated including surgical removal of the infection source, broad-spectrum antibiotics, and anticoagulation therapy.^[1,5,35]^ In our case, we immediately started heparinization and systemic antibiotics, and we delayed surgical treatment because some reported cases successfully recovered with interval appendectomy.^[23]^ However, the abdominal pain was not relieved; therefore, a laparoscopic appendectomy was performed.

The role and duration of anticoagulation therapy in patients with mesenteric venous thrombosis is still controversial,^[5,36]^ but anticoagulation therapy is reported to lower the recurrence of thrombosis and the mortality rate.^[1,36]^ The recommended total duration of anticoagulant therapy is at least 3 to 6 months,^[1,37,38]^ although a longer duration is suggested if a thrombophilic condition is identified.^[39]^ In our case, the patient received intravenous unfractionated heparin during the hospital stay and then apixaban for 3 months after discharge. He was successfully treated without other complications or a recurrence of thrombosis.

Apixaban, a factor Xa inhibitor, is 1 of the non-vitamin K antagonist oral anticoagulants (NOACs), and is a safer alternative for the prevention of venous thromboembolism than vitamin K antagonists (e.g., warfarin) in adults.^[40]^ NOACs do not need specific monitoring and have fewer complications (e.g., a lower incidence of major bleeding) than warfarin.^[38,40]^ Moreover, apixaban has been reported to be effective in both the treatment and prevention of deep vein thrombosis (DVT) and for the postoperative prevention of DVT.^[41]^ Although the use of NOACs in pediatric patients has limited data, recent studies found similar concentration-related effects between adults and children.^[42]^ Furthermore, clinical outcomes in adult studies and pharmacological properties suggest that apixaban may have particular advantages for children.^[42]^ There are currently several ongoing studies.^[43–45]^ In the present case, we prescribed apixaban because the patient was a previously healthy adolescent and due to the risk of postoperative bleeding, and to improve anticoagulant therapy adherence. He was successfully treated without other complications or a recurrence of thrombosis.

## 4 Conclusion

Mesenteric venous thrombosis is a rare but possible complication of acute appendicitis in previously healthy pediatric patients. Its non-specific clinical characteristics can make an early diagnosis difficult. Mesenteric venous thrombosis should be considered when prolonged unexplained abdominal pain, fever, or elevated liver enzyme levels are concomitant in a patient with appendicitis (whether before or after surgery). Appendicitis is the most common cause of pediatric surgical emergencies;^[46]^ therefore, clinical awareness and concern for mesenteric venous thrombosis is warranted for physicians.

## Author contributions

**Conceptualization:** Mi-Jung Lee.

**Data curation:** Se Yong Jung, In Geol Ho.

**Investigation:** Mi-Jung Lee.

**Supervision:** Moon Kyu Kim.

**Writing – original draft:** Seo Hee Yoon.

**Writing – review & editing:** Moon Kyu Kim.
